# Tanycytic transcytosis inhibition disrupts energy balance, glucose homeostasis and cognitive function in male mice

**DOI:** 10.1016/j.molmet.2024.101996

**Published:** 2024-07-22

**Authors:** Manon Duquenne, Eleonora Deligia, Cintia Folgueira, Cyril Bourouh, Emilie Caron, Frank Pfrieger, Markus Schwaninger, Ruben Nogueiras, Jean-Sébastien Annicotte, Monica Imbernon, Vincent Prévot

**Affiliations:** 1 Univ. Lille, Inserm, CHU Lille, Laboratory of Development and Plasticity of the Neuroendocrine Brain, Lille Neuroscience & Cognition, UMR_S1172, EGID, DISTALZ, Lille, France; 2 CIMUS, Universidade de Santiago de Compostela-Instituto de Investigación Sanitaria, Santiago de Compostela, 15782, Spain; 3 CIBER Fisiopatología de la Obesidad y Nutrición (CIBERobn), 15706, Spain; 4 Univ. Lille, Inserm, CHU Lille, Institut Pasteur de Lille, U1011-EGID, F-59000 Lille, France; 5 Centre National de la Recherche Scientifique, Universite de Strasbourg, Institut des Neurosciences Cellulaires et Integratives, 67000 Strasbourg, France; 6 Institute of Experimental and Clinical Pharmacology and Toxicology, Center of Brain, Behavior and Metabolism, University of Lübeck, Lübeck, Germany; 7 Univ. Lille, Inserm, CHU Lille, Institut Pasteur de Lille, U1167 - RID-AGE - Facteurs de risque et déterminants moléculaires des maladies liées au vieillissement, F-59000 Lille, France

**Keywords:** Tanycytes, Blood–brain barrier, Blood-cerebrospinal fluid barrier, Transports, Hypothalamus, Normal-weight central obesity

## Abstract

**Objectives:**

In Western society, high-caloric diets rich in fats and sugars have fueled the obesity epidemic and its related disorders. Disruption of the body-brain communication, crucial for maintaining glucose and energy homeostasis, arises from both obesogenic and genetic factors, leading to metabolic disorders. Here, we investigate the role of hypothalamic tanycyte shuttles between the pituitary portal blood and the third ventricle cerebrospinal fluid in regulating energy balance.

**Methods:**

We inhibited vesicle-associated membrane proteins (VAMP1-3)-mediated release in tanycytes by expressing the botulinum neurotoxin type B light chain (BoNT/B) in a Cre-dependent manner in tanycytes. This was achieved by injecting either TAT-Cre in the third ventricle or an AAV1/2 expressing Cre under the control of the tanycyte-specific promoter iodothyronine deiodinase 2 into the lateral ventricle of adult male mice.

**Results:**

In male mice fed a standard diet, targeted expression of BoNT/B in adult tanycytes blocks leptin transport into the mediobasal hypothalamus and results in normal-weight central obesity, including increased food intake, abdominal fat deposition, and elevated leptin levels but no marked change in body weight. Furthermore, BoNT/B expression in adult tanycytes promotes fatty acid storage, leading to glucose intolerance and insulin resistance. Notably, these metabolic disturbances occur despite a compensatory increase in insulin secretion, observed both in response to exogenous glucose boluses in vivo and in isolated pancreatic islets. Intriguingly, these metabolic alterations are associated with impaired spatial memory in BoNT/B-expressing mice.

**Conclusions:**

These findings underscore the central role of tanycytes in brain-periphery communication and highlight their potential implication in the age-related development of type 2 diabetes and cognitive decline. Our tanycytic BoNT/B mouse model provides a robust platform for studying how these conditions progress over time, from prediabetic states to full-blown metabolic and cognitive disorders, and the mechanistic contribution of tanycytes to their development. The recognition of the impact of tanycytic transcytosis on hormone transport opens new avenues for developing targeted therapies that could address both metabolic disorders and their associated cognitive comorbidities, which often emerge or worsen with advancing age.

## Highlights

• Blockade of tanycytic transcytosis abrogates access of blood-borne signals to the hypothalamus.
• Obstructing tanycytic shuttles promotes intra-abdominal fat accumulation and carbohydrate consumption.
• Expression of botulinum toxin in tanycytes alters peripheral lipid homeostasis.
• Hindering tanycytes transports promotes glucose intolerance and insulin resistance.
• Impairing tanycytic transcytosis leads to normal-weight central obesity and cognitive alterations.

## Graphical abstract

**Figure:**
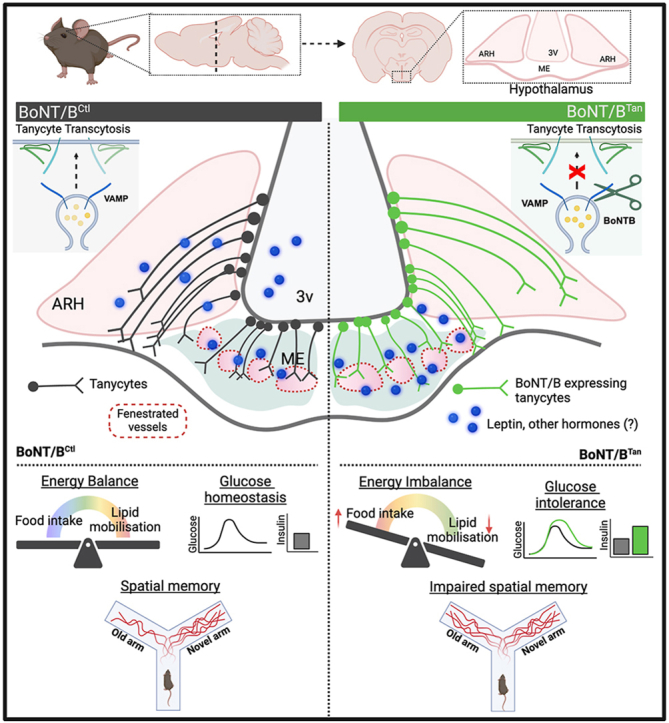

## Abbreviations

ACCa: acetyl-CoA carboxylase
ARH: arcuate nucleus of the hypothalamus
AVV: adeno-associated virus
BBB: blood–brain barrier
BoNT/B: Botulinum toxin type B light chain
CPT1-A: protein expression of carnitine palmitoyl transferase 1-A
CSF: cerebrospinal fluid
CVO: circumventricular organ
DREADD: Designer Receptor Exclusively Activated by Designer Drugs
EGFP: enhanced green fluorescent protein
ER: endoplasmic reticulum
FACS: fluorescent activated cell sorting
FAS: fatty acid synthase
GSIS: glucose-stimulated insulin secretion
HOMA-IR: homeostatic model assessment of insulin resistance
HSL: hormone-sensitive lipase
LPL: lipoprotein lipase protein
SNARE: Soluble N-ethylmaleimide-sensitive-factor Attachment protein REceptor
T2D: Type 2 diabetes
VAMP: vesicle-associated membrane protein
3V: third ventricle

## 1 Introduction

Since the mid-20th century, the development of the processed food industry in Western society has created a high-caloric eating culture, which, together with a sedentary lifestyle, resulted in an epidemy of obesity and a plethora of obesity-related disorders such as cardiovascular diseases, type 2 diabetes (T2D), steatohepatitis, cancer, and neurodegenerative disorders [1]. Nutrient sensing mechanisms that maintain glucose and energy homeostasis involve body-brain communication by peripheral humoral and neuronal components, that will inform the brain about peripheral energy status and nutrient availability [[2], [3], [4]]. Impairment of this communication due to both obesogenic and genetic factors can lead to the development of metabolic disorders. In recent decades, advances in neuroendocrinology have enabled the development of new strategies to treat metabolic diseases. These approaches aim to restore communication between the body and the brain that is often disrupted in conditions of energy excess [[5], [6], [7]]. A deeper understanding of cells and molecules mediating communication between the periphery and the brain is essential for developing more effective and long-lasting therapies for metabolic diseases.

Within the tuberal region of the hypothalamus, the median eminence serves as a key central hub for integrating signals related to energy balance by functioning as a circumventricular organ (CVO) [8]. The median eminence is the structure closing the ventral aspect of the third ventricle (3V) adjacent to the arcuate nucleus of the hypothalamus (ARH) which is a major site of the metabolic brain containing the melanocortin system [6]. The median eminence houses a dense capillary network with fenestrated endothelium, allowing blood-borne cues about the body's energy status to freely enter the median eminence and the ventromedial ARH [ [9,10]]. However, the extent of passive molecule diffusion is limited, and the fenestration of capillary loops reaching the ARH is finely regulated according to energy status [11].

Tanycytes are specialized ependymoglial cells that line the 3V floor. They form tight junctions that prevent blood-borne molecules from reaching deeper hypothalamic structures via the cerebrospinal fluid (CSF) [[12], [13], [14]]. Fine processes of these cells establish direct contact with fenestrated vessels, controlling the bidirectional exchange of molecules between the bloodstream and the hypothalamus [14,15]. This exchange delivers neurohormones into the bloodstream from neurons that regulate the anterior pituitary's activity and permits blood-borne molecules to reach their target neurons in the hypothalamus [[16], [17], [18], [19]]. Moreover, tanycytes themselves are central to intercellular communication processes within hypothalamic circuits that regulate energy homeostasis. They sense certain nutrients such as glucose and communicate their concentration to neurons [20]. Selectively manipulating tanycytic activity using the DREADD technology or genetically altering their viability has profound consequences on energy homeostasis and circulating metabolic hormone levels [[21], [22], [23]]. This is particularly relevant given that the conduit of blood-borne metabolic hormones into the hypothalamus by tanycytes is impaired in conditions of energy surplus [24,25]. Our previous research indicates that the disruption of tanycyte shuttles may impair vesicular trafficking within these cells that is crucial for transcytosis processes [16].

To explore the importance of SNARE-dependent vesicle trafficking in tanycytes for metabolic regulation, we employed Cre recombinase-mediated expression of clostridial botulinum neurotoxin serotype B light chain (BoNT/B) in tanycytes in adult mice. Our findings demonstrate that the blockade of tanycytic transcytosis alone is sufficient to abrogate the access of blood-borne signals such as leptin into the mediobasal hypothalamus. This manipulation impairs glucose homeostasis in mice fed on a standard diet: it promotes intraabdominal fat deposition, glucose intolerance, hyperinsulinemia and impaired insulin sensitivity, particularly in the context of moderate overweight. Additionally, it heightens sensitivity to obesogenic diets.

## 2 Materials and methods

### 2.1 Animals

All C57Bl/6J male mice were housed under specific pathogen-free conditions in a temperature-controlled room (21–22 °C) with a 12h light/dark cycle, 40% humidity and *ad libitum* access to food and water. Tg(CAG-BoNT/B,EGFP)U75-56Fwp/J (*BoNT/B-EGFP*^loxP-STOP-loxP^; JAX Stock No. 018056) mice were generated as previously described [26]. Transgenic model mice were bred and genotyped in-house to generate experimental animals. All experiments were performed in 8–20 weeks-old mice and with the approval of the Institutional Ethics Committees for the Care and Use of Experimental Animals of the University of Lille and the French Ministry of National Education, Higher Education and Research (APAFIS#2617–2015110517317420 v5), following the guidelines outlined by the European Union Council Directive of September 22, 2010 (2010/63/EU).

### 2.2 Stereotaxic delivery of TAT-Cre and AAVs

Tanycyte-selective recombination in BoNT/B-EGFP^loxP-STOP-loxP^ was performed with Cre/LoxP system in isoflurane-anesthetized 8-week old mice by stereotaxic infusion of a TAT-Cre fusion protein (1.27 mg ml^−1^) [27], AAV1/2-Dio2Cre (0.5 × 10^10^ genomic particles μl^−1^) [23] or a 1:1 mix of AAV1/2-Dio2Cre (2.5 × 10^9^ genomic particles μl^−1^) and AAV1/2-CMV > EGFP:WPRE (2.5 × 10^9^ genomic particles μl^−1^) (VectorBuilder, VB010000-9394npt). Both TAT-Cre or vehicle were stereotaxically infused into the third ventricle (2 μL; at 0.2 μl/min; anteroposterior, −1.7 mm; midline, 0 mm; dorsoventral, −5.6 mm), whereas viral AAV1/2 vectors were administrated in the lateral ventricle (1 μL; at 0.3 μl/min; LV; anteroposterior, −0.3 mm; midline, ±1 mm; dorsoventral, −2.5 mm) 1 and 3 weeks before experiments for TAT-Cre and virus injections, respectively, as previous described [16].

### 2.3 Isolation of hypothalamic tanycytes using fluorescence-activated cell sorting

Median eminence from TAT-Cre-injected TdTomato^loxP−STOP-loxP^ or BoNT/B-EGFP^loxP-STOP-loxP^ mice and AAV1/2 Dio2Cre- or AAV1/2 Dio2GFP-injected BoNT/B-EGFP^loxP-STOP-loxP^ mice were microdissected, and enzymatically dissociated using Papain Dissociation System (Worthington Papain, Lakewood, NJ) to obtain single-cell suspensions as described [28]. FACS experiments were performed using an EPICS ALTA Cell Sorter Cytometer device (Beckman Coulter, Brea, CA, USA). The cell sort decision was based on measurements of tdTomato fluorescence (excitation, 561 nm; detection: bandpass. 675 ± 20 nm) or EGFP fluorescence (EGFP: excitation: 488 nm; 50 mW; detection: EGFP bandpass 530/30 nm, autofluorescence bandpass 695/40 nm) by comparing cell suspensions from non-infected brain sites (the cortex) and infected brain sites (median eminence). For each TAT-Cre-injected TdTomato animal, 4,000 tdTomato-positive and -negative cells were isolated, while for each AAV1/2 Dio2Cre- or vehicle-injected BoNT/B-EGFPloxP-STOP-loxP mice, between 200 and 3000 EGFP- positive and negative cells were sorted directly into 10 μL extraction buffer: 0,1% Triton® X-100 (Sigma–Aldrich) and 0.4 U/μl RNaseOUT™ (Thermo Fisher, Waltham, MA, USA).

### 2.4 Quantitative RT-PCR analyses

For gene expression analyses of FACS-sorted tanycytes, mRNAs were reverse transcribed using SuperScript® III Reverse Transcriptase (Life Technologies, Carlsbad, CA, USA) and a followed by linear preamplification step using the TaqMan® PreAmp Master Mix Kit protocol (P/N 4366128, Applied Biosystems, Foster City, CA, USA). Real-time PCR was carried out on QuantStudio™ 3 Real-Time PCR System, using exon-boundary-specific TaqMan® Gene Expression Assays: botulinum toxin serotype-B light chain (Custom-made 2169611 A6-APAAJ3N), Vamp1 (Mm00772307_m1), Vamp2 (Mm01325243_m1)***,*** Vamp3 (Mm05693405_s1), Dio2 (Mm00515664_m1) and Ppp1r1b (Mm00454892_m1). Control housekeeping genes: r18S (18S-Hs99999901_s1); ACTB (Actb-Mm00607939_s1).

### 2.5 Indirect calorimetry study

Mice were individually housed and acclimatized to the cages for 48h before experimental measurements in a 12h light/dark cycle and an ambient temperature of 22 ± 1 °C. Mice fed a chow diet were placed in metabolic cages 6 weeks after AAV1/2 Dio2Cre-induced recombination. Mice fed a high-fat diet (HFD) were placed in metabolic cages 10 weeks after AAV1/2 Dio2Cre injection, which was 4 weeks after they began consuming the HFD. Mice were analyzed for total energy expenditure, oxygen consumption, carbon dioxide production, and food intake using calorimetric cages (Labmaster, TSE Systems GmbH, Bad Homburg, Germany) [29]. Fat oxidation was calculated as described before [30,31], using energy expenditure, oxygen consumption, and carbon dioxide production. Mice were monitored daily for body weight and body composition at the beginning and the end of the experiment.

### 2.6 Hormone measurements

Both serum and microdialysate leptin concentrations in BoNT/B^Ctl^ and BoNT/B^Tan^ mice were measured by Leptin Elisa Kit (Mouse/Rat Leptin ELIOS – Quantikine, MOB00, R&D systems). Serum noradrenaline content was measured using a noradrenaline Elisa kit (BA-E5200, Immusmol). Both measurements were performed following the manufacturer's instructions. Plasma insulin levels from the glucose tolerance test were measured with a Mouse Insulin Elisa Kit (Mercodia, 10-1247-01).

### 2.7 Lipid measurements

Serum cholesterol (Spinreact, no. 1001093), triglycerides (Spinreact, no. 1001310), and non-esterified fatty acid levels (Wako, nos. 436–91995, 434–91795) were measured by colorimetry following kits instructions, and plates were read on a ThermoScientific Multiskan GO spectrophotometer.

### 2.8 Immunoblot analyses of liver and white adipose tissue

Tissues were homogenized in a TissueLyser II (Qiagen) in cold RIPA buffer (200 mM Tris/HCl pH 7.4, 130 mM NaCl, 10 (v/v glycerol, 0.1% (v/v) SDS, 1% (v/v) Triton X-100, 10 mM MgCl2) with anti-proteases and anti-phosphatases (Sigma–Aldrich). Lysates were centrifuged for 30 min at 18,000g and 4 °C. Total protein lysates were separated on SDS–polyacrylamide gel electrophoresis (SDS–PAGE), electrotransferred to polyvinylidene difluoride membranes and then probed with the following antibodies: hormone-sensitive lipase/HSL (1 1,000; Abcam, catalog no. ab45422); phospho-HSL (Ser660) (1:1,000; Cell Signaling Technology, catalog no. 4126); Acc (1:1,000; Millipore, catalog no. 04–322); phospho-Acc (Ser79) (1:1,000; Cell Signaling Technology, catalog no. 3661); lipoprotein lipase (1:1,000; H-53, Sant Cruz Biotechnology, catalog no. sc-32885); FAS antibody (1:5,000 Abcam, catalog no. ab128870); CPT1A: (Abcam, Cambridge, UK, ab128568); and β-actin (1:5,000; Sigma–Aldrich, catalog no. A2228), after incubation of membranes with 5% BSA blocking buffer. Proteins were detected using horseradish peroxidase–conjugated secondary antibodies (Dako, no. PI-2000). Specific immunolabelling was visualized using chemiluminescence following the manufacturer's instructions (Pierce ECL Western Blotting Substrate, ThermoScientific), and values expressed relative to β-actin.

### 2.9 Glucose tolerance tests

After overnight fasting (12 h), the first glycemia measurement was taken (time 0) and then mice received an intraperitoneal glucose injection (1.5 mg glucose g^−1^ body weight). Glycemia was subsequently measured at intervals of 15, 30, 60, 90, 120, and 150 min after glucose administration (OneTouch Verio glucometer). Blood samples were collected with capillaries from the tail at times 0, 15, and 30 min to detect insulin levels. Following centrifugation, plasma was collected and stored at −80 °C until hormone measurement.

### 2.10 Insulin tolerance test

Mice were subjected to 6-h fasting after the onset of the light phase, and after basal blood glucose levels were measured (OneTouch Verio glucometer) they received an intraperitoneal injection of insulin (0.75 UI kg–1 body weight). Glycaemia was monitored 15, 30, 45, 60, 120, and 150 min after insulin administration.

### 2.11 Homa-IR

Homeostatic model assessment for insulin resistance (HOMA-IR) was calculated by using fasting plasma insulin (FPI, μU/L) multiplied by fasting plasma glucose (FPG, mg/dL) divided by a constant (HOMA-IR score = FPI × FPG/405), as previously described [32].

### 2.12 Mouse pancreatic islet isolation and culture

Each of the ten mice used for islet isolation was fully anesthetized and sacrificed by cervical dislocation. The mouse was laid with the abdomen facing up and the skin was sterilized with 70% ethanol. An incision was performed around the upper abdomen to expose the pancreas and common bile duct. The pancreas was infused with type V collagenase (1.5 mg/ml) via the common bile duct. After perfusion, the pancreas was removed, collected in a 50 ml tube containing 2 ml of enzyme collagenase, and digested in a water bath at 37 °C for 8–10 min [33,34]. Enzymatic digestion was stopped by adding cold Hanks’ Balanced Salt solution containing 1% albumin. A density gradient was then performed with polysucrose 1,132/1,108/1,096/1,069/1,000 (Mediatech) to get a high-purity fraction of islets. Islets showed >90% purity (endocrine versus exocrine tissue). They were cultured in RPMI-1640 medium (Sigma Aldrich) containing 2 g of glucose, supplemented with 10% FBS and 1% P/S for 18 h before treatment. For insulin secretion tests, ∼30 islets were exposed to either 2.8- or 20-mM glucose in Krebs–Krebs-Ringer-bicarbonate HEPES buffer containing 0.5% FA-free bovine serum albumin (BSA). Secreted insulin was measured 1 h later using an Ultrasensitive Insulin ELISA kit (Mercodia, 10-1132-01). Insulin content was measured after lysis in a buffer containing 75% of ethanol and 1.5% HCl using mouse insulin ELISA kit. Data are expressed relative to total insulin content.

### 2.13 RNA extraction, measurement and profiling of pancreatic islets

Total RNA was extracted from islets using an RNeasy Plus Micro Kit (Qiagen) following the manufacturer's recommendations. mRNA levels were measured after reverse transcription by quantitative PCR with reverse transcription using FastStart SYBR Green master mix (Roche), following the manufacturer's recommendations and with gene-specific oligonucleotides as detailed in Supplementary Table 1. Expression levels were normalized to cyclophilin mRNA and expressed using formula 2–ΔCt.

### 2.14 Immunofluorescence of pancreatic sections

Immunofluorescence was performed as described previously [33]. After antigen retrieval using citrate buffer (Sigma), formalin-fixed pancreatic sections (5 μm) were incubated with either anti-insulin (1:1,000; Agilent, catalog no. A0564) or anti-glucagon primary antibodies (1:1,000; Sigma–Aldrich, catalog no. G2654) and AlexaFluor-594-conjugated goat anti-guinea pig (1:500 Molecular Probes, catalog no. A-11076) and AlexaFluor-488-conjugate goat anti-mouse antibodies (1:500; Thermo Fisher Scientific, catalogue no. A-11001). Images were processed for morphometry using ImageJ software by an observer blind to experimental groups.

### 2.15 Immunohistochemistry and analysis

*In fresh frozen tissue*. Adult mice were sacrificed by decapitation at lights-on in fed state (for p-STAT3 and MECA32). Brains were harvested before being embedded directly in Tissue Tek (Sakura®) and frozen fresh. Coronal sections 20 μm thick were post-fixed with a 2% paraformaldehyde solution for 1 h and processed for immunofluorescence as previously described (Bouret et al., 2012) using primary antibodies directed against STAT3 phosphorylated at Tyr705 (p-STAT3, 1:1000; #9131, Cell Signaling Technology) and against plasmalemma vesicle-associated protein (clone MECA32; 1:500; 550563, BD Parmingen). Double immunofluorescence images were acquired using an Apotome Axio.Z2 microscope (AxioCam MRm camera, Zeiss). Slides were then coded to conceal treatment groups, and p-STAT3 immunoreactive (IR) cells were counted on eight representative sections per animal. MECA-32 immunoreactive vessels are visualized in the superficial plexus of the outer zone of the median eminence, but some MECA-32 immunoreactive vessels form intra-infundibular capillary loops. The total number of fenestrated loops and p-STAT3 immunopositive cells was assessed over the entire rostrocaudal surface of the median eminence and arcuate nucleus in each animal (8 representative median eminence sections per animal). The average number of immunopositive cells or vessels per area was then compared between groups.

*In paraformaldehyde-fixed tissue*. Mice were intracardially perfused with 4% paraformaldehyde in PBS 0.01M pH 7.4. The brains were harvested and post-fixed in the same fixative solution overnight. Thereafter, brains were cryoprotected in 30% sucrose-PBS, frozen on dry ice, and cut into two series of 40-μm thick coronal sections using a stage cryomicrotome (Leica®, Germany). Free-floating brain sections were blocked for 1h in 0.4% Triton-0.5% BSA in PBS 0.01M pH7.4, and then they were then incubated overnight with: goat polyclonal anti-vimentin antibody (1:500, sc-7557, Santa-Cruz) and rabbit polyclonal anti-GFP antibody (Rabbit 1:1,000 A6455, Invitrogen, ThermoFisher Scientific); or with rabbit polyclonal anti-cleaved caspase 3 antibody (Rabbit 1:200, 9661, Cell Signaling) at 4 °C under mild agitation. Brain sections were washed 3 times per 5 min in PBS before incubation with secondary antibodies. Sections were incubated in a mix of the corresponding secondary antibodies (1:500 Invitrogen, ThermoFisher Scientific Donkey Anti-Rabbit IgG (H + L), Alexa Fluor™ 647 ref: A31573, Donkey anti-Goat IgG (H + L) Cross-Adsorbed Secondary Antibody, Alexa Fluor™ 647 ref: A11055, Jackson ImmunoResearch Laboratories Inc. Donkey Anti-Chicken Alexa Fluor® 488 ref: 703-545-155). All secondary antibodies were diluted in PBS and incubated with sections for 90 min at room temperature. Nuclei were counterstained by incubating sections with DAPI (1:5,000 D9542, Sigma Aldrich) for 5 min at RT. All labelled sections were mounted onto Superfrost glass slides and images acquired using an Apotome Axio.Z2 microscope (AxioCam MRm camera, Zeiss) or a STELLARIS Confocal microscope platform (Leica).

### 2.16 Leptin sensitivity assay

Individually housed mice were subjected to fasting for 6h before the onset of the dark phase as previously described [35,36]. Three hours before the refeeding, 2 groups of mice (both BoNT/B^Ctl^ and BoNT/B^Tan^) were challenged with leptin. A group received either murine leptin (3 mg/kg^−1^; Harbor-UCLA Medical Center) or vehicle (PBS pH 8.0) intraperitoneally. To check the ICV effect of leptin administration, a cannula (Plastic One) was stereotaxically placed in the lateral ventricle (anteroposterior, −0.3 mm; midline, ±1 mm; dorsoventral, −2.5 mm) and leptin (2 μg in 2 μl Harbor-UCLA Medical Center) or vehicle (2 μl of PBS pH 8.0) was administrated to a second group of mice. Body weight and food intake were monitored before,12h, and 24h after leptin administration.

### 2.17 In vivo microdialysis

A microdialysis cannula (CMA8 High Cut-off, 100 kDa, 1 mm membrane length; CMA microdialysis) was stereotaxically implanted in the mediobasal hypothalamus (anteroposterior, −1.3 mm; lateral, −0.3 mm; ventral, −6.1 mm) in BoNT/B^Ctl^ and BoNT/B^Tan^ isoflurane-anesthetized mice, maintained at 37 °C core body temperature using a thermostatically controlled electrical blanket. The cannula was then perfused at 2 μl min–1 with sterile artificial CSF (ACSF; CNS Perfusion Fluid: NaCl 147 mmol l–1, KCl 2.7 mmol l–1, CaCl2 1.2 mmol l–1 and MgCl2 0.85 mmol l–1; CMA) using a microinjection pump (CMA 402, CMA). Following stabilization for 45 min, two basal dialysates of 20 min were collected. Leptin (i.p., 3 mg kg–1 in PBS pH 8.0; Harbor-UCLA Medical Center) was administered to mice and six dialysates of 20 min recovered. Dialysates were placed in a fraction collector (CMA 820) during the experiment and immediately stored at −80 °C until analysis. At the end of the experiment, mice were decapitated and brains were stored immediately in fresh 4% paraformaldehyde. Microtome brain sections (40 μm) were counterstained with DAPI to verify probe location. Only mice in which the probe was positioned between anteroposterior −1.2 and −2.3 were included in analyses.

### 2.18 Behavioral assessment

Visuospatial short-term memory and novelty preference were tested using the Y-maze [37]. The Y-maze consists of three white wooden arms (24.0 cm × 6.5 cm x 15 cm) on the floor and surrounded with visual cues on the walls. Mice were placed in the start arm, facing the end of this arm, and were allowed to explore the maze for 10 min while one arm was blocked (novel arm). Consequently, mice were placed in their home cage for 2 min before being allowed to explore all three arms for 5 min. The trajectories of the mice were recorded using EthoVision video tracking equipment and software (Noldus Bv, Wageningen, The Netherlands). The primary outcome measure was the percentage of time spent in the novel arm compared to the familiar arms, indicative of the subject's spatial memory and exploratory behaviour. The time spent in the novel arm and latency to enter the novel arm were compared between mice. The Y-maze test was performed in the morning (9:00).

### 2.19 Statistics

Results are given as means ± s.e.m. Samples or animals were excluded when their values were outside ±2 s d., or when an objective experimental failure was observed; studies were not formally randomized and investigators were not blind to the treatment group, except when mentioned. To test whether populations followed a Gaussian distribution, a normality test was performed (Kolgomorov–Smirnov test for n = 5–7, Shapiro–Wilk test for n ≥ 7). For normal distributions, one or two-sided unpaired t-tests were used to compare two populations; for multiple-comparison tests, one or two-way ANOVA followed by Tukey's post hoc multiple-comparison test was used (unless otherwise indicated in the figure legends). For non-Gaussian distributions, Mann–Whitney tests were used to compare two populations, and Kruskal–Wallis followed by Dunn's post hoc test for multiple comparisons. The ANCOVA analysis was performed using the NIDDK Mouse Metabolic Phenotyping Centers online tools (MMPC, www.mmpc.org/). Data analysis was performed using GraphPad Prism Software v.8.0 (version 8.4.2; GraphPad Software, La Jolla, California, USA). The threshold for significance was P < 0.05.

## 3 Results

### 3.1 Botulinum neurotoxin type B expression in adult hypothalamic tanycytes promotes body weight gain while increasing carbohydrate consumption

**Figure 1:**
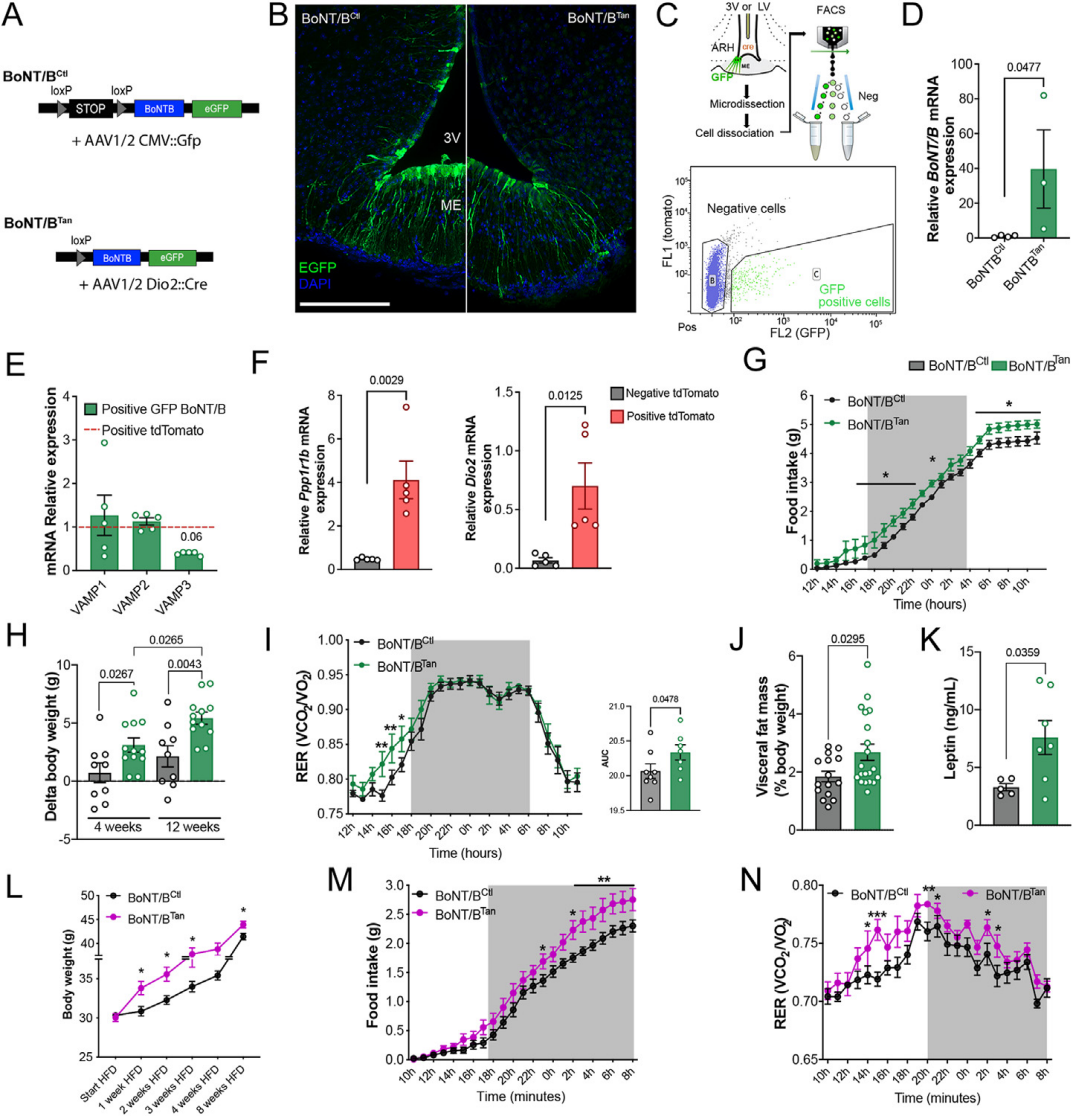
**Alteration of vesicular trafficking in median eminence tanycytes promotes body weight gain.** (**A**) Schematics for the Cre-dependent induction of BoNT/B in tanycytes after the injection of recombinant TAT-Cre or vehicle into the third ventricle (3V), or AAV1/2-Dio2-Cre or vehicle in the lateral ventricle (LV) of BoNT/B-EGFP^loxP-STOP-loxP^ mice. (**B**) Representative photomicrographs of EGFP immunopositive median eminence tanycytes in BoNT/B^Ctl^ and BoNT/B^Tan^ mice at 4 weeks (AAV1/2 -injected mice). Scale bar, 200 μm. (**C**) Diagram and gating strategy for the sorting of EGFP-positive putative tanycytes following AAV1/2-Dio2-Cre infusion into the LV of BoNT/B-EGFP^loxP-STOP-loxP^ mice. (**D**) mRNA levels of Botulinum neurotoxin light chain B mRNA in EGFP-positive FACS-sorted cells from ME and ARH BoNT/B^Tan^ and BoNT/B^Ctl^ explants at 4 weeks (AAV1/2- injected mice). One-tailed unpaired *t*-test; t = 2.051, df = 5, n = 4 and 3 mice. Botulinum neurotoxin light chain B mRNA was undetextale in EGFP-negative FACS-sorted cells. (**E**) Expression of VAMP1, VAMP2 and VAMP3 mRNA in EGFP-positive FACS-sorted cells of BoNT/B^Tan^ mice (n = 5) at 4 weeks (TAT-Cre or vehicle injected mice), normalized over the same mRNA expression (Value = 1) in Tomato-positive FACS-sorted cells of TdTomato^Tan^ mice (n = 5). Two-tailed unpaired t-test, *VAMP1* t = 0.5214, df = 8, p = 0.6162; VAMP2 t = 0.8441, df = 8, p = 0.4231; VAMP3 t = 2.183, df = 8, p = 0.0606. (**F**) Expression of the tanycytic markers *Ppp1r1b* coding DARPP-32 and *Dio2* in tomato-positive and negative cells isolated by FACS. Two-tailed unpaired t-test, *Ppp1r1b* t = 4.221, df = 8; *Dio2* t = 3.204, df = 8. (**G**) Curves representing 24h of cumulative food intake in BoNT/B^Ctl^ (n = 7) and BoNT/B^Tan^ (n = 8) mice at 4 weeks (AAV1/2-injected mice). Two-way ANOVA, Interaction p = 0.6123, F_(23,299)_ = 0.8901; Time p < 0.0001, F (23,299) = 707.3; Subjects p = 0.0266, F _(1,13)_ = 6.250. LSD post-hoc test, 16.00h p = 0.0411; 17.00h p = 0.0414; 18.00h p = 0.0164; 19.00h p = 0.0159; 20.00h p = 0.0112; 21.00h p = 0.0324; 22.00h p = 0.0357; 4.00h p = 0.041; 5.00h p = 0.0361; 6.00h p = 0.013; 7.00h p = 0.0154; 8.00h p = 0.0103; 9.00h p = 0.0108; 10.00h p = 0.0095 and 11.00h p = 0.0275. (**H**) Body weight change of BoNT/B^Ctl^ (n = 9) and BoNT/B^Tan^ (n = 12) mice at 4 and 12 weeks after starting the experiments (AAV1/2-injected mice). Values are expressed in grams (gr).Two-way ANOVA, Time p = 0.0008, F _(1, 16)_ = 16.84; subjects p = 0.0041, F _(1, 22)_ = 10.29; Šídák's multiple comparisons test, BoNT/B^Ctl^ 4 weeks versus BoNT/B^Ctl^ 12 weeks p = 0.254. (**I**) Curves representing 24h of respiratory exchange ratio (RER) in BoNT/B^Ctl^ (n = 7) and BoNT/B^Tan^ (n = 8) mice at 4 weeks (AAV1/2 -injected mice). Two-way ANOVA, Interaction p = 0,5424, F _(23, 299)_ = 0.9417; Time p = <0.0001, F _(23, 299)_ = 69.28; Subjects p = 0.0927, F _(1, 13)_ = 3.293. LSD post-hoc test, 15.00h p = 0.0039; 16.00h p = 0.0081; 17.00h p = 0.0179. The area under the curve (AUC), one-tailed unpaired t-test, t = 1.797, df = 13. (**J**) Visceral fat mass expressed as a percentage of the body weight in BoNT/B^Ctl^ (n = 14) and BoNT/B^Tan^ (n = 12) at 12 weeks (AAV1/2- injected mice). Two-tailed Mann–Whitney test. Values are expressed as percentages calculated over the body weight. (**K**) Circulating leptin levels of BoNT/B^Ctl^ (n = 5) and BoNT/B^Tan^ (n = 7) mice at 12 weeks (TAT-Cre or vehicle infusion into the 3V). Two-tailed unpaired t-test, t = 2.422, df = 1. (**L**) Body weight follow-up in BoNT/B^Ctl^ (n = 6) and BoNT/B^Tan^ (n = 5) mice fed with a high-fat diet (HFD) for 4 weeks, at 4 weeks (AAV1/2-injected mice). Two-way ANOVA, Interaction p = 0.0821, F (4, 88) = 2.143; Time p < 0.0001, F (1,688, 37,13) = 73.62; Subjects p = 0.0228, F (1, 22) = 5.990. The two-stage linear step-up procedure of Benjamini, Krieger and Yekutieli, at the start of the HFD, p = 0.2245; 1-week HFD p = 0.0153; 2-week HFD p = 0.0121; 3-week HFD p = 0.0292 and 4-week HFD p = 0.0994. (**M**) Curves representing 24h cumulative food intake in BoNT/B^Ctl^ (n = 6) and BoNT/B^Tan^ (n = 6) mice at 8 weeks after the beginning of the experiment (AAV1/2-injected mice) and 4 weeks of HFD. Two-way ANOVA, Interaction p < 0.0001, F _(23, 230)_ = 3.125; Time p < 0.0001, F _(23, 230)_ = 385.4; Subject p = 0.0672, F _(1, 10)_ = 4.213. LSD post-hoc test, 23.00h p = 0.0452; 1.00h p = 0.0276; 2.00h p = 0.0042; 3.00h p = 0.0022; 4.00h p = 0.0052; 5.00h p = 0.0034; 6.00h p = 0.0042; 7.00h p = 0.0064; 8.00h p = 0.0066; 9.00h p = 0.0111. (**N**) Curves representing 24h RER in BoNT/B^Ctl^ (n = 6) and BoNT/B^Tan^ (n = 6) mice at 8 weeks after the beginning of the experiment (AAV1/2-injected mice) and 4 weeks of HFD. Two-way ANOVA, Interaction p = 0.5955, F (23, 230) = 0.9024; Time p < 0.0001, F (23, 230) = 17,87; Subject p = 0.0169, F (1, 10) = 8,185. LSD post-hoc test, 14.00h p = 0.0442; 15.00h p = 0.0002; 17.00h p = 0.0067; 20.00h p = 0.0379; 2.00h p = 0.0432 and 3.00h p = 0.0229. Data are expressed as mean ± SEM. p < 0.05 values are indicated in the figures.

To explore the specific function of tanycytes in transporting metabolic hormones from the bloodstream to the brain, we employed a mouse model where we inhibited vesicular transport within tanycytes using botulinum toxin light chain B (BoNT/B). BoNT/B hinders exocytosis in neurons and glial cells by cleaving vesicle-associated membrane proteins 1 and 2 (VAMPs), which facilitate vesicular fusion and transport [26,38]. VAMP3, another target of BoNT/B, is involved both in endocytosis and exocytosis processes [39,40]. For conditional cell-specific BoNT/B expression, we used males from a transgenic mouse line in which BoNT/B and EGFP can be expressed in a Cre-dependent manner [26]. To induce Cre expression and thus BoNT/B specifically in tanycytes of these mice, we injected either TAT-Cre into the 3rd ventricle [16] or of an AAV1/2 vector expressing the Cre recombinase under the control of the tanycytes-specific human deiodinase 2 (DIO2) promoter into the lateral ventricle [23]. Control littermates were injected with an AAV1/2 vector expressing EGFP. Control- and Cre-injected animals are hereafter referred to as BoNT/B^Ctl^ and BoNT/B^Tan^ mice, respectively (Figure 1A). The viral strategy selectively targeted tanycytes in the tuberal region of the hypothalamus (Figure 1B). Isolation of tanycytes using fluorescent-activated cell sorting (FACS) from median eminence-ARH microdissected explants (Figure 1C) showed strong induction of BoNT/B transcripts in BoNT/B^Tan^ mice in contrast to BoNT/B^Ctl^ littermates, which did not show any signal (Figure 1D). Transcripts for VAMP1-3 were readily detected in FACS-sorted tomato-positive tanycytes isolated from tdTomato^loxP−STOP-loxP^ mice following TAT-Cre delivery into the third ventricle (Figure 1E) that express tanycytic markers, including *Ppp1r1b* and *Dio2* (Figure 1F). Notably, as expected the presence of BoNT/B did not affect the expression of VAMP1 and VAMP2 genes remained unchanged in targeted tanycytes. However, there was a trend toward downregulation of VAMP3 transcripts compared to tanycytes from tdTomato mice (Figure 1E). Crucially, the expression of BoNT/B in tanycytes did not change their morphology (Figure 1B) nor did it induce cell death. No nuclear fragmentation, which would indicate apoptosis, was observed in the tanycytic cell body layer adjacent to the third ventricle (Supplementary Fig. 1A), and no cleaved caspase 3 signal was detected in the cell nuclei lining the wall and the floor of the third ventricle in mice expressing BoNT/B at 4 and 12 weeks after injection of the Cre-expressing viruses (Supplementary Figs. 1B and C).

Four weeks after Cre-mediated genetic recombination and intake of a chow diet, mice expressing BoNT/B in tanycytes exhibited increased food intake (Figure 1G), which was associated with a slight increase in body weight (Figure 1H, Supplementary Fig. 2A) compared to BoNT/B^Ctl^ littermates. Surprisingly, indirect calorimetric analyses revealed elevated carbohydrate metabolism at the end of the resting period, as indicated by a higher respiratory exchange ratio (RER, Figure 1I), along with increased energy expenditure and ambulatory activity (Supplementary Figs. 2B–C). ANCOVA analysis [[41], [42], [43]] revealed no significant interaction between total body mass (TBM) and energy expenditure (EE) across genotypes (p = 0.51), and when adjusted for TBM, there was no significant difference in EE between control and BoNT/B-expressing mice (p = 0.4). However, the observed difference in EE was significantly associated with the difference in TBM between groups (p = 0.046), suggesting that the altered EE in BoNT/B-expressing mice is primarily due to changes in body mass rather than a direct effect of genotype on energy metabolism. At 12 weeks post recombination, BoNT/B^Tan^ mice exhibited a more consistent net body weight gain compared to their control littermates (Figure 1H, Supplementary Fig. 2A). Interestingly, this occurred despite the absence of actual body weight differences between genotypes (Supplementary Fig. 2A). However, the BoNT/B^Tan^ mice showed two notable metabolic changes: increased accumulation of visceral fat (Figure 1J) and higher circulating levels of leptin (Figure 1K). Altogether the BoNT/B^Tan^ mice appear to develop a “normal-weight obesity” phenotype, i.e, intra-abdominal accumulation of fat with slight overweight or no marked changes in body weight [44,45].

When animals were placed on a high-fat diet (HFD) four weeks after the beginning of the experiment using AAV-mediated Cre recombination, body weight gain markedly accelerated in BoNT/B^Tan^ mice, with significant changes occurring already within two weeks after initiating this diet (Figure 1L). As observed with the chow diet (Figure 1G, I), indirect calorimetry at four weeks on the HFD showed that BoNT/B^Tan^ mice exhibited increased food intake (Figure 1M), accompanied by increased use of carbohydrates as the primary energy source during both resting and active periods (Figure 1N). However, no marked differences were observed in energy expenditure or ambulatory activity (Supplementary Figs. 2D–E). Altogether these data show that impairing SNARE-dependent vesicle release from tanycytes enhances food intake and body weight. These changes were possibly caused by disruption of the feedback loops between peripheral tissues and the brain, leading to miscommunication regarding energy balance.

### 3.2 BoNT/B expression in tanycytes blocks blood-borne leptin transport into the hypothalamus

**Figure 2:**
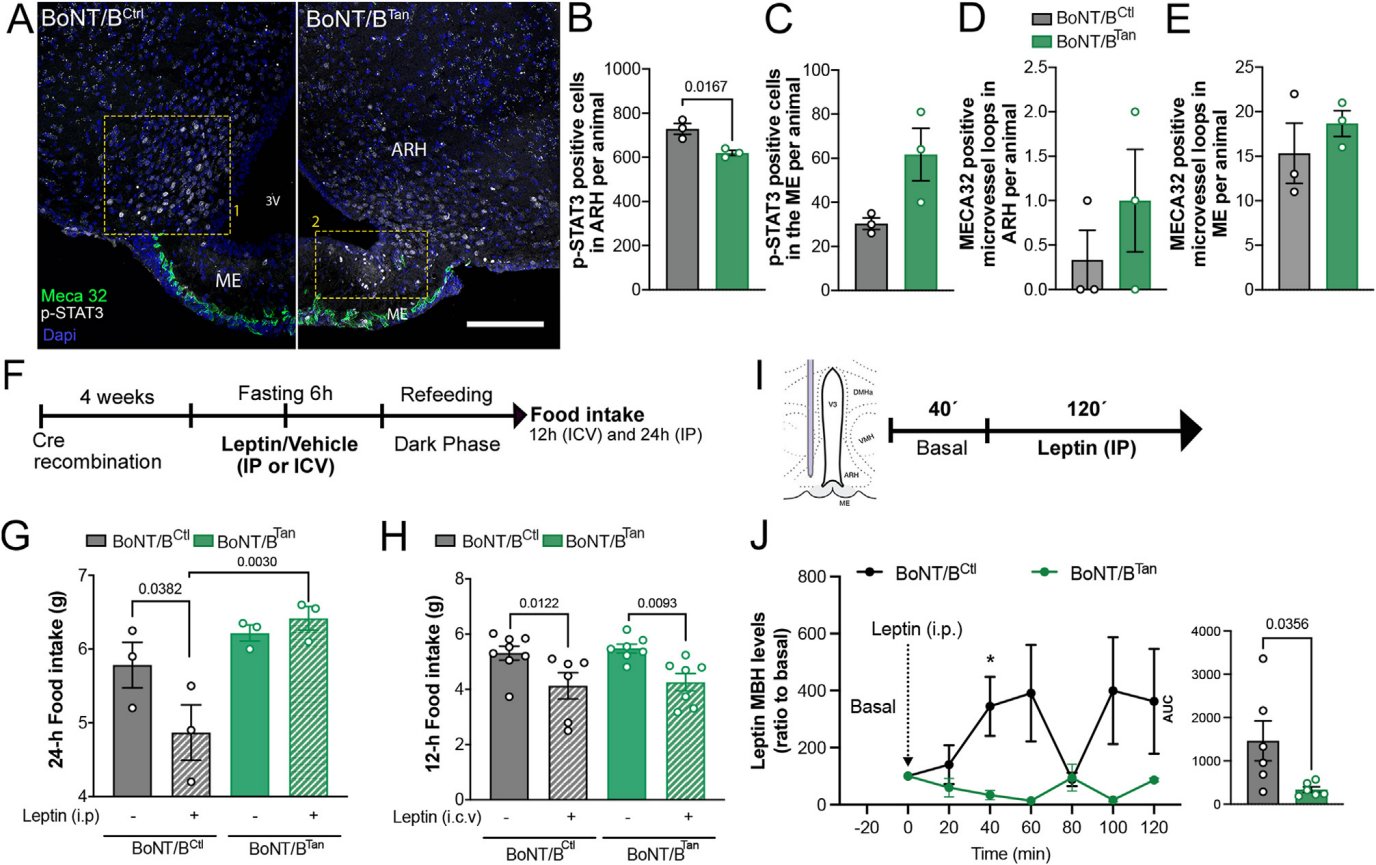
**Blockade of exocytosis in ME tanycytes impairs leptin sensitivity and its transport into the medio basal hypothalamus.** (**A**) Representative photomicrographs of phosphorylated STAT3 (p-STAT3) immunopositivity (white dots) in median eminence and medio basal hypothalamus cells and MECA32 immunopositive microvessel loops (green) in BoNT/B^Ctl^ and BoNT/B^Tan^ mice at 4 weeks after the beginning of the experiment (AAV1/2-injected mice). Yellow dotted insets one and two delimitate the ARH and ME respectively. Scale bar, 200 μm. (**B**,**C**) Quantification of p-STAT3 immunopositive cells in the ARH (**B**) and ME (**C**) of BoNT/B^Ctl^ (n = 3) and BoNT/B^Tan^ (n = 3) mice at 4 weeks (AAV1/2-injected mice). Two-tailed unpaired t-test. In ARH (**B**), t = 3.961, df = 4; ME (**C**), p = 0.0617, t = 2.574, df = 4). (**D**,**E**) Quantification of the number of MECA32 immunopositive microvessel loops in the ARH (**D**) and ME (**E**) in in BoNT/B^Ctl^ (n = 3) and BoNT/B^Tan^ (n = 3) mice 4 weeks after Cre-induced genetic recombination. Two-tailed unpaired t-test. In ARH (**D**), p = 0.3739, t = 1.000, df = 4; ME (**E**), p = 0.4165 t = 0.9054, df = 4. (**F**) Protocol of leptin tolerance test (i.p. 3 mg/kg or i.c.v. 1 mg/kg) in BoNT/B^Ctl^ and BoNT/B^Tan^ mice 12 weeks after the beginning of the experiment (TAT-Cre- or vehicle-injected mice). (**G**) Cumulative food intake 24 h after leptin or vehicle injection in BoNT/B^Ctl^ (n = 3) and BoNT/B^Tan^ (n = 3) mice. Two-way ANOVA, Interaction p = 0.0653, F _(1, 8)_ = 4.557; Treatment p = 0.2079, F _(1, 8)_ = 1.877; Subject p = 0.0053, F _(1, 8)_ = 14.38. LSD post hoc test, BoNT/B^Ctl^_Veh_ vs. BoNT/B^Tan^_Veh_ p = 0.2751 and BoNT/B^Tan^_Veh_ vs. BoNT/B^Tan^_Leptin_ p = 0.6034. (**H**) Cumulative food intake 12 h after leptin (i.c.v. 1 mg/kg) or vehicle injection in BoNT/B^Ctl^ (n = 8) and BoNT/B^Tan^ (n = 7). Two-way ANOVA, Interaction p = 0.9514 F _(1, 24)_ = 0.00379; Treatment p = 0.0006 F _(1, 24)_ = 15.34; Subject p = 0.6308 F _(1, 24)_ = 0.237. LSD post hoc test, BoNT/B^Ctl^_Veh_ vs. BoNT/B^Tan^_Veh_ p = 0.6908 and BoNT/B^Ctl^_Leptin_ vs. BoNT/B^Tan^_Leptin_ p = 0.7740. (**I**) Schematic diagram of the stereotactic implantation of the microdialysis probe in the mediobasal hypothalamus (MBH) and the protocol of the same experiment, that investigates exogenous leptin central transport in BoNT/B^Ctl^ and BoNT/B^Tan^ mice 4 weeks after the beginning of the experiment (AAV1/2-injected mice). (**J**) Leptin concentrations in the MBH extracellular fluid collected by microdialysis every 20 min following intraperitoneal leptin (t, 0 min) injection (3 mg/kg) in BoNT/B^Ctl^ (n = 5) and BoNT/B^Tan^ mice (n = 5). Mixed-effects analysis, Time p = 0.3901, F (0.8537, 5.265) = 0.7959; Subject p = 0.0423, F (1, 12) = 5.159; Interaction p = 0.1708, F (6, 37) = 1.614. LSD post hoc test, 40min p = 0.0387. AUC, two-tailed unpaired t-test t = 2.427, df = 10. Data are expressed as mean ± SEM. p < 0.05 values are indicated in the figures. (For interpretation of the references to color/colour in this figure legend, the reader is referred to the Web version of this article.)

Increased food intake (Figure 1G) concomitant with elevated adiposity and circulating levels of leptin in BoNT/B^Tan^ mice (Figure 1J,K) raises the possibility that these animals may be developing hypothalamic resistance to circulating leptin. This could be caused by defective leptin transport across the blood–brain barrier into the CSF via tanycytes, as seen in various animal models at early stages of diet-induced obesity [16,24,46]. First, to test whether this phenomenon occurs under physiological conditions, we assessed endogenous STAT3 activation in the ARH at the specific time of the day when lights are switched on, after overnight feeding, when circulating leptin levels are at their highest [47]. This paradigm has previously been shown to be linked to endogenous LepR activation [11,48]. Four weeks after Cre-induced recombination, the number of P-STAT-3 immunoreactive cells in the ARH was diminished by about 30% in BoNT/B^Tan^ mice when compared to BoNT/B^Ctl^ littermates (Figure 2A,B). In contrast, a trend towards an increased number of p-STAT3-immunoreactive cells was observed in the median eminence of BoNT/B^Tan^ mice compared to control littermates, although this difference did not reach statistical significance (Figure 2C). This suggests that in BoNT/B^Tan^ mice, circulating leptin extravasates from the fenestrated capillaries of the median eminence but appears to remain trapped within the median eminence parenchyma. There, it may activate local LepR-expressing cells without being transported into the hypothalamus. In support of this, examination of the fenestral diaphragms of median eminence endothelial cells using antibodies to MECA-32 showed that the number of fenestrated capillary loops in the median eminence and the vmARH were comparable in BoNT/B^Tan^ and control littermates (Figure 2A,D and 2E). Finally, twelve weeks after Cre-induced recombination, we subjected BoNT/B^Tan^ mice as well as their control littermates to intraperitoneal (i.p.) or intracerebroventricular (i.c.v.) injection of exogenous leptin and measured food intake 24h and 12h later, respectively (Figure 2F–H). While exogenous leptin injected i.c.v. directly into the lateral ventricle reduced feeding in both groups of mice efficiently (Figure 2H), exogenous leptin injected intraperitoneally showed this effect only in BoNT/B^Ctl^ mice, but not in BoNT/B^Tan^ mice (Figure 2G). This confirmed that BoNT/B^Tan^ mice are sensitive to central leptin but resistant to peripheral leptin suggesting a block of the tanycytic transport of blood-borne leptin into the hypothalamus. To further explore the impact of BoNT/B expression in tanycytes on blood-borne leptin transport into the ARH, we stereotaxically implanted microdialysis probes into the mediobasal hypothalamus of both BoNT/B^Ctl^ and BoNT/B^Tan^ mice (Figure 2I). Leptin levels in the hypothalamus were monitored in dialysates over time. Forty minutes after administering an intraperitoneal bolus of leptin, we observed leptin transport into the mediobasal hypothalamus in BoNT/B^Ctl^ mice, following a kinetic pattern that was not observed in BoNT/B^Tan^ littermates. In the latter, leptin levels remained close to the detection limit of the approach (Figure 2J). Altogether, these results unequivocally demonstrate that tanycytic transcytosis is required for the transport of blood-borne leptin into the hypothalamus.

### 3.3 BoNT/B expression in hypothalamic tanycytes alters peripheral lipid homeostasis

**Figure 3:**
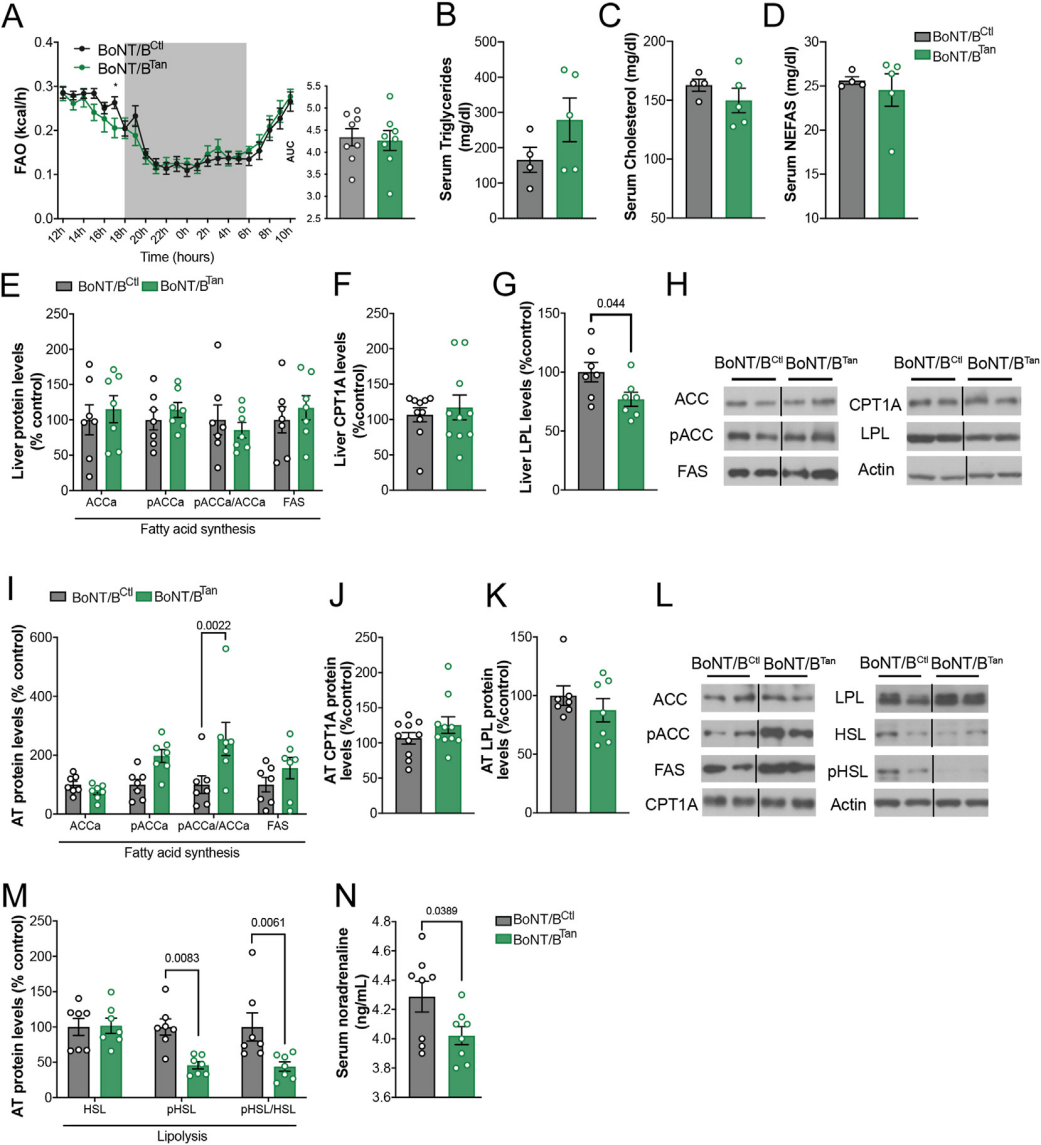
**BoNT/B expression in tanycytes reduces lipid mobilization in adipose tissue.** (**A**) Curves representing 24h fatty acid oxidation (FAO) in BoNT/B^Ctl^ (n = 7) and BoNT/B^Tan^ (n = 8) mice, 4 weeks after the beginning of the experiment (AAV1/2-injected mice). Two-way ANOVA, Interaction p = 0.3634, F_(23,322)_ = 1.082; Time p < 0.0001, F _(4.788, 67.03)_ = 35.71; Subjects p = 0.8217, F _(1,14)_ = 0.05273. LSD post-hoc test, 17h p = 0.0483). AUC, one-tailed unpaired t-test, p = 0.4069, t = 0.2400, df = 14. (**B**–**D**) Circulating levels of triglycerides (**B**), cholesterol (**C**) and non-esterified fatty acids (NEFAs, **D**) of BoNT/B^Ctl^ (n = 4) and BoNT/B^Tan^ (n = 5) mice at 12 weeks (TAT-Cre- or vehicle-injected mice). Two-tailed unpaired t-test; triglycerides, p = 0.1832, t = 1.477, df = 7; cholesterol, p = 0.3396, t-test, t = 1.025, df = 7; NEFAs, p = 0.626, t test, t = 0.5096, df = 7. (**E**–**G**) Production levels of selected proteins involved in fatty acid (FA) synthesis in liver (**E**), carnitine-palmitoyl transferase A (CPT1A) (**F**) and lipoprotein lipase (LPL) (**G**), from BoNT/B^Ctl^ (n = 10) and BoNT/B^Tan^ (n = 10) mice at 12 weeks (TAT-Cre- or vehicle-injected mice). In **E**, one-way ANOVA, p = 0.0005, F = 5.803. Šídák's multiple comparisons test ACCa BoNT/B^Ctl^ vs. ACCa BoNT/B^Tan^ p = 0.9638, t = 0.5811; pACCa BoNT/BCtl vs. pACCa BoNT/B^Tan^ p = 0.9721 t = 0.5405; pACCa/ACCa BoNT/BCtl vs. pACCa/ACCa BoNT/B^Tan^ p = 0.9227 t = 0.7239 and FAS BoNT/B^Ctl^ vs. FAS BoNT/B^Tan^ p = 0.9456 t = 0.6528. In **F**, two-tailed unpaired t-test, p = 0.6178, t = 0.5078, df = 18). In **G**, two-tailed unpaired t-test, t = 2.250, df = 12). (**H**) Representative immunoblots of proteins from panels **E**–**G**. (**I**–**M**) Expression levels of selected proteins involved in FA synthesis in adipose tissue (**I**), CPT1A (**J**), LPL (**K**) and proteins involved in lipolysis (**M**), from BoNT/B^Ctl^ (n = 7) and BoNT/B^Tan^ (n = 7) mice at 12 weeks (TAT-Cre- or vehicle-injected mice). In **I**, one-way ANOVA, p = 0.0007, F = 4.431. Šídák's multiple comparisons test ACCa BoNT/B^Ctl^ vs. ACCa BoNT/B^Tan^ t = 05776, p = 0.9646; pACCa BoNT/B^Ctl^ vs. pACCa BoNT/B^Tan^ p = 0.0927, t = 2.331; pACCa/ACCa BoNT/B^Ctl^ vs. pACCa/ACCa BoNT/B^Tan^ t = 3.705; and FAS BoNT/B^Ctl^ vs. FAS BoNT/B^Tan^ p = 0.5632, t = 1.338. In **J**, two-tailed unpaired p = 0.2124, t test, t = 1.293, df = 18. In K), two-tailed Mann Whitney test, p = 0.535). In **M**, one-way ANOVA, p = 0.0005. Šídák's multiple comparisons test, HSL BoNT/B^Ctl^ vs. HSL BoNT/B^Tan^ p = 0.9995, t = 0.1026; pHSL BoNT/B^Ctl^ vs. pHSL BoNT/B^Tan^ t = 3.214 and pHSL/HSL BoNT/B^Ctl^ vs. pHSL/HSL BoNT/B^Tan^ t = 3.329. (**L**) Representative immunoblots of proteins in (**I–K**, **M**). (**N**) Circulating noradrenaline levels of BoNT/B^Ctl^ (n = 8) and BoNT/B^Tan^ (n = 8) mice at 12 weeks (TAT-Cre- or vehicle-injected mice). One-tailed Mann Whitney test. Data are expressed as mean ± SEM. p < 0.05 values are indicated in the figures.

Despite the higher visceral fat mass and leptinemia observed in BoNT/B^Tan^ mice compared to their BoNT/B^Ctl^ littermates (Figure 1J-K), no significant differences were found in either fatty acid oxidation (Figure 3A) or serum levels of triglycerides (Figure 3B), cholesterol (Figure 3C), and non-esterified free fatty acids (NEFAS) between the two groups of mice (Figure 3D). To delve deeper into lipid metabolism, we examined the lipolysis and lipogenesis pathways in both the liver in visceral adipose tissue of BoNT/B^Ctl^ and BoNT/B^Tan^ littermates using Western blot analyses.

In the liver, BoNT/B expression in tanycytes did not affect markers of de novo lipogenesis, including the total protein levels and phosphorylation of acetyl-CoA carboxylase (ACCa) and fatty acid synthase (FAS) (Figure 3E,H). Also, protein expression of carnitine palmitoyl transferase 1-A (CPT1-A), an indicator of fatty acid oxidation, remained unchanged (Figure 3F,H). However, there was a notable reduction in lipoprotein lipase protein (LPL) levels (Figure 3G, H), suggesting a decrease in hepatic lipid uptake.

In the epididymal white adipose tissue, BoNT/B^Tan^ mice showed higher ratio of phosphorylated ACCa (pACC)/ACCa, indicating a decrease in fatty acid synthesis (Figure 3I,L). No differences were observed in the protein levels of the fatty acid oxidation marker CPT1A (Figure 3J,L) or in the lipid uptake marker LPL (Figure 3K,L). However, BoNT/B^Tan^ mice exhibited reduced phosphorylation of hormone-sensitive lipase (HSL) compared to control mice (Figure 3M, 3L), suggesting a decrease in triglyceride mobilization, possibly due to lower noradrenaline levels, an activator of HSL-mediated lipolysis (Figure 3N) [49,50]. Overall, these results suggest that the blockade of tanycyte transcytosis in BoNT/B^Tan^ mice may reduce lipid synthesis and mobilization in the white adipose tissue. This could be due to the incapacity of leptin to reach the ARH and promote peripheral lipid mobilization [51], as well as hindered signaling to the sympathetic nervous system via the melanocortin system [52], as suggested by the decreased noradrenalin levels in the BoNT/B^Tan^ mouse model (Figure 3N). Nonetheless, it is also possible that other hormones are involved, their passage impeded, thereby affecting lipid metabolism in BoNT/B^Tan^ mice.

### 3.4 Expression of BoNT/B in tanycytes promotes glucose intolerance and insulin resistance associated with pancreatic beta cell compensation

**Figure 4:**
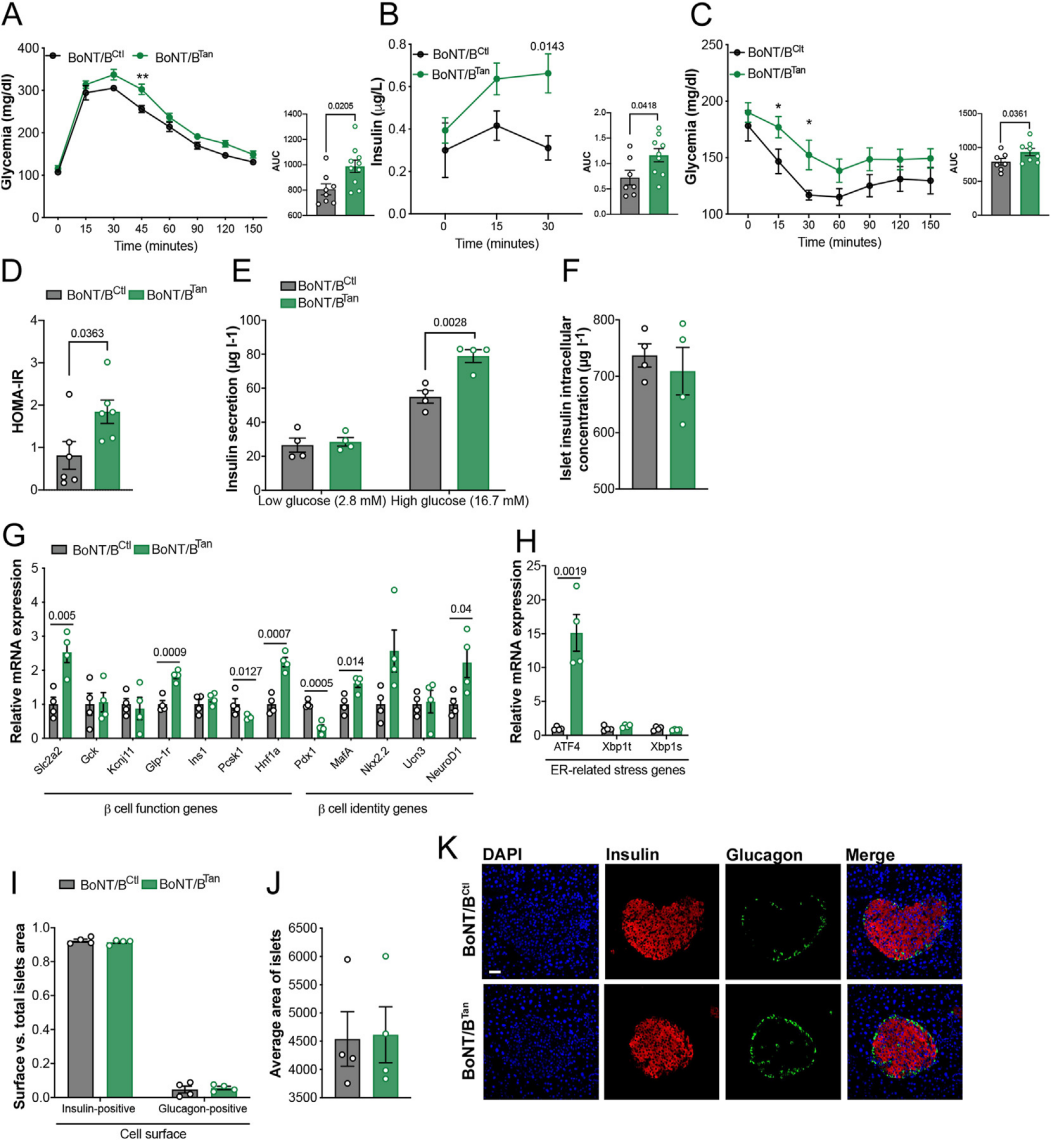
**Expression of BoNT/B in tanycytes impairs glucose metabolism triggering compensatory mechanisms in pancreatic beta cells**. (**A**) Curve representing glycaemia during a glucose tolerance test in BoNT/B^Ctl^ (n = 7) and BoNT/B^Tan^ (n = 8) mice at 12 weeks (TAT-Cre- or vehicle-injected mice). Two-way ANOVA, Interaction p = 0.2853, F _(7,112)_ 1.243; Time p = <0.0001, F _(7, 112)_ = 249.5; Treatment p = 0,0167, F _(1, 16)_ = 7.136. Bonferroni's multiple comparisons test, 45′ p = 0,0006, 120′ p = 0,0376). AUC, two-tailed unpaired t-test, t = 2.693, df = 16. (**B**) Serum insulin levels 0, 15 and 30 min after intraperitoneal delivery of glucose (2 g/kG) during the glucose tolerance test in **A**. Two-way ANOVA, Interaction p = 0.1142, F _(2,28)_ = 2.347; Time p = 0.0138, F _(2, 28)_ = 5.011; Treatment p = 0.0364, F _(1,14)_ = 5.351. Šídák's multiple comparisons test, 0min p = 0.8174; 15min p = 0.1925. AUC, two-tailed unpaired t-test, t = 2.286, df = 14. (**C**) Glycaemia during an insulin tolerance test in BoNT/B^Ctl^ (n = 7) and BoNT/B^Tan^ (n = 8) mice at 12 weeks (TAT-Cre- or vehicle-injected mice). Two-way ANOVA; Time p = <0.0001, F _(6, 78)_ = 26.36; Subject p = 0.0864, F _(1, 13)_ = 3.441. Uncorrected Fisher's LSD, 15min p = 0.0377; 30min p = 0.0150. AUC, one-tailed unpaired t-test, t = 1.955, df = 13. (**D**) Homa-IR, two-tailed unpaired t-test, t = 2.417, df = 10). (**E**) Insulin secretion in a static incubation of pancreatic islets isolated from BoNT/BCtl (n = 4) and BoNT/B^Tan^ (n = 4) mice, at 12 weeks (TAT-Cre- or vehicle-injected mice). Two-way ANOVA, Interaction p = 0.0091, F _(1,12)_ = 9,645; Glucose concentration p = <0.0001, F _(1, 12)_ = 121.9; Subject p = 0,0034, F _(1, 12)_ = 13.21. Šídák's multiple comparisons test, Low glucose _(2,8 mM):_BoNT/B^Ctl^ vs. Low glucose _(2,8 mM)_:BoNT/B^Tan^ p = 0.9995; Low glucose _(2,8 mM)_:BoNT/B^Ctl^ vs. High glucose _(16,7 mM)_:BoNT/B^Ctl^ p = 0.0007; Low glucose _(2,8 mM)_:BoNT/B^Tan^ vs. High glucose _(16,7 mM)_:BoNT/B^Tan^ p = <0.0001. (**F**) Intracellular insulin content of pancreatic islets isolated from BoNT/B^Ctl^ (n = 4) and BoNT/B^Tan^ (n = 4) mice at 12 weeks (TAT-Cre- or vehicle-injected mice). Two-tailed unpaired t-test, p = 0.5756, t = 0.5918, df = 6. (**G**,**H**) Relative mRNA expression levels of β-cell function and identity (**G**) and endoplasmic reticulum (ER) stress markers (**H**) in pancreatic islets isolated from BoNT/B^Ctl^ (n = 4) and BoNT/B^Tan^ (n = 4) mice at 12 weeks (TAT-Cre- or vehicle-injected mice). Unpaired t test, two-stage step-up (Benjamini, Krieger, and Yekutieli). In **G**, *Gck* p = 0.8919; *Kcnj11* p = 0.7457; *Ins1* p = 0.3688; *Pcsk1* p = 0.07637; *Nkx2.2* p = 0.0555 and *Ucn3* p = 0.8395. In **H**, *Xbp1t* p = 0.1444; *Xbp1s* p = 0.2788. (**I**) Ratio between insulin-positive and glucagon-positive area to total islet surface area in BoNT/B^Ctl^ (n = 4) and BoNT/B^Tan^ (n = 4) mice at 12 weeks (TAT-Cre- or vehicle-injected mice). Two-way ANOVA, Interaction p = 0.516, F _(1, 12)_ = 0,4799; Insulin/Glucagon p = < 0.0001, F _(1, 12)_ = 4700; Subjects p = 0.9949, F _(1, 12)_ = 4.304e-005. Šídák's multiple comparisons test, Insulin-positive:BoNT/B^Ctl^ vs. Insulin-positive:BoNT/B^Tan^ p = 0.9609 and Glucagon-positive:BoNT/B^Ctl^ vs. Glucagon-positive:BoNT/B^Tan^ p = 0.9588. (**J**) Average surface area of pancreatic islets from **I**. Two-tailed unpaired t-test, p = 0.9182, t = 0.1071, df = 6. (**K**) Representative photomicrographs representing nuclei (blue), insulin (red) and glucagon (green) in isolated pancreatic islets from BoNT/B^Ctl^ and BoNT/B^Tan^ mice at 4 weeks (TAT-Cre- or vehicle-injected mice) in **I** and **J**. Scale bar, 50 μm. Data are expressed as mean ± SEM. p < 0.05 values are indicated in the figures. (For interpretation of the references to color/colour in this figure legend, the reader is referred to the Web version of this article.)

At 12 weeks after BoNT/B induction in tanycytes, BoNT/B^*Tan*^ mice exhibited impaired tolerance to exogenous glucose (Figure 4A). Monitoring of glucose-stimulated insulin release monitoring revealed heightened insulin secretion in BoNT/B^*Tan*^ mice compared to control BoNT/B^Ctl^ littermates, suggesting a beginning of insulin resistance (Figure 4B). An insulin tolerance test further confirmed this observation (Figure 4C). Additionally, insulin sensitivity and the homeostatic model assessment of insulin resistance (HOMA-IR) were found to be elevated in BoNT/B^Tan^ mice compared to control mice (Figure 4D), indicating a prediabetic, insulin-resistant state in BoNT/B^Tan^ overweight mice. To assess potential changes in pancreatic function in mice expressing BoNT/B in tanycytes, we examined glucose-stimulated insulin secretion (GSIS) in pancreatic islets isolated from both BoNT/B^Tan^ and BoNT/B^Ctl^ littermates 12 weeks after Cre-mediated recombination. Consistent with the prediabetic, insulin-resistant phenotype observed in BoNT/B^Tan^ mice, isolated islets demonstrated heightened insulin secretion in response to 20 mM glucose, but similar insulin content compared to those from control BoNT/B^Ctl^ littermates (Figure 4E,F), suggesting the activation of compensatory mechanisms in β cells following insulin resistance in these animals [53]. Gene expression profiling of β-cells from BoNT/B^Ctl^ and BoNT/B^Tan^ islets revealed notable transcriptional alterations in markers of β-cell function and endoplasmic reticulum (ER) stress. In β-cells from BoNT/B^Tan^ mice compared to controls, the heightened GSIS was accompanied by upregulation of mRNA levels in genes implicated in glucose sensing and insulin secretion, including *Slc2a2* and *Glp-1r.* Conversely, there was a decrease in expression of the proinsulin processing gene *Pcsk1* (Figure 4G). Additionally, differences were noted in the expression of genes associated with pancreatic islet identity and function, such as reduced expression of *Pdx1* involved in β-cell maturation and survival [54], and elevated levels of *Hnf1a, MafA* and *NeuroD1* genes, all contributing to β-cell identity (Figure 4G). Notably, β cells from BoNT/B^Tan^ mice exhibited elevated expression of the early onset ER-stress marker *Atf4* (Figure 4H), suggesting that the increased insulin secretion observed in these mice following insulin resistance may trigger an ER unfolded protein response and ER stress. This response is typically associated with impaired β-cell function under chronic high glucose [55]. Despite this, no differences were observed yet in surface area (Figure 4I), size (Figure 4J), or morphology (Figure 4K) of pancreatic islets from BoNT/B^Tan^ compared to those from BoNT/B^Ctl^ mice.

Overall, tanycytic transcytosis appears to be an essential part of the central regulation of pancreatic function, whose blockade results in glucose intolerance, insulin resistance and the disruption of β-cell function and identity independently of pancreatic islet morphology.

### 3.5 Blunting transcytosis in tanycytes alters spatial working memory

**Figure 5:**
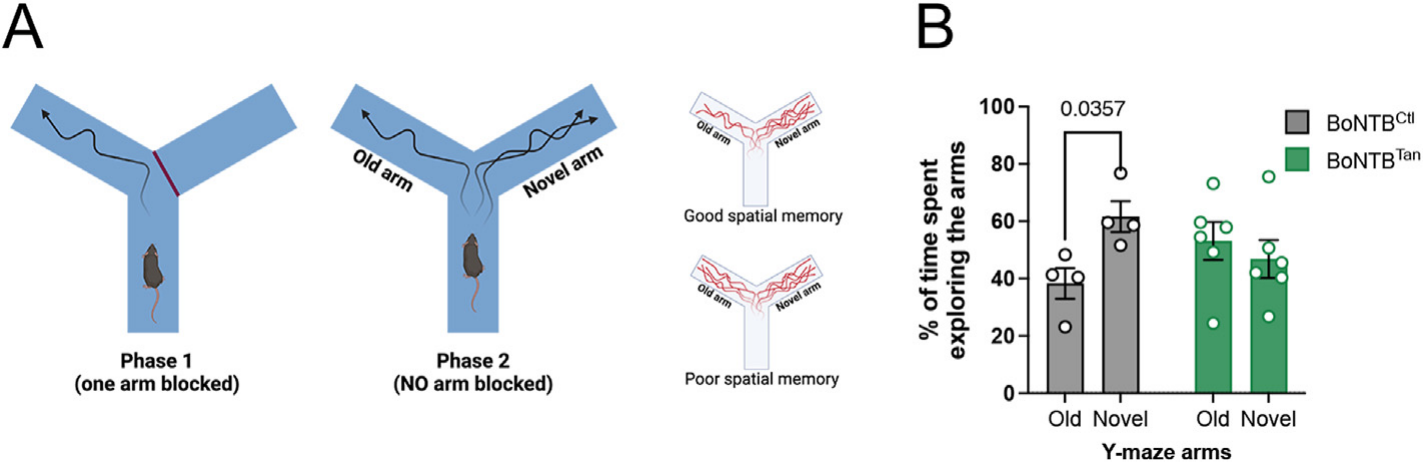
**BoNT/B expression in tanycytes impairs spatial working memory.** (**A**) Diagram representing the Y-maze test. In phase one, only one arm is accessible for the mice to explore. In phase 2, both arms of the maze are accessible, challenging the spatial memory of the mice by quantifying the time they spent in each arm. (**B**) Percentage of time that BoNT/B^Ctl^ (n = 4) and BoNT/B^Tan^ (n = 6) spent in the arms during phase 2 of the Y-maze test, at 12 weeks (AAV1/2 -infused mice) in the lateral ventricle. One-tailed unpaired t-test; BoNT/B^Ctl^ old-arm vs BoNT/B^Ctl^ new-arm, t = 2.140, df = 6; BoNT/B^Tan^ old-arm vs BoNT/B^Tan^ new-arm, t = 0.05559, df = 10, p = 0.2952. Two-way ANOVA, Interaction p = 0.0384, F (1,16) = 5.092; Space p = 0.2131, F (1,16) = 1.682; Genptype P > 0,9999, F (1,16) = 0. Uncorrected Fisher's LSD, BoNT/BTanOld vs BoNT/BTan Novel p = 0.4590. Data are expressed as mean ± SEM. p < 0.05 values are indicated in the figures.

Next, we aimed to evaluate potential early cognitive effects of the metabolic changes observed in our mouse model, focusing on hippocampus-dependent behaviors that are often the first to show impairment in age-related cognitive decline [56,57]. We employed the Y-maze test, which specifically assesses spatial working memory, a form of short-term memory, and exploratory behavior, both of which heavily rely on hippocampal function. Twelve weeks after viral injection, BoNT/B^Ctl^ mice subjected to this test showed normal behavior, spending more time exploring the newly accessible arm than the previously explored arms (Figure 5). In contrast, BoNT/B^Tan^ mice spent an equal length of time in all arms, suggesting impaired spatial memory (Figure 5). These results suggest that disrupting tanycytic shuttling, and consequently body-brain communication, may progressively impair spatial working memory, contributing to the onset of cognitive decline in mice, particularly in the context of metabolic disturbances.

## 4 Discussion

Over the last decade, tanycytes have been proposed as the conduit for peripheral information into the hypothalamus, aiding in the regulation of body homeostasis. To achieve this, they actively transport blood-borne signals across the blood-cerebrospinal fluid (CSF) barrier, which they themselves form [24]. Using the selective expression of BoNT/B in tanycytes to inhibit vesicular trafficking, we provide compelling evidence to show that tanycytic transcytosis, which shuttles not only circulating hormones and peripheral signals but also nutrients into the brain, is essential for maintaining energy homeostasis and cognitive processes.

Previous studies have demonstrated that the conditional ablation or impaired function of median eminence tanycytes results in a phenotype similar to that observed in our model, characterized by increased adiposity, insulin resistance, elevated caloric intake, and leptin resistance [22,58]. However, these studies also revealed compromised barrier properties in the median eminence, allowing peripheral cues to bypass the tanycytic barrier and tanycyte-mediated changes in barrier architecture induced by energy status [11] or circadian rhythms [59]. Through a more targeted approach, we can now elucidate the specific role of tanycytic transport in modulating energy balance. By expressing BoNT/B in tanycytes, we impede the transport of blood-borne molecules transported via tanycytic transcytosis into the ARH, and deprive BBB-protected ARH neurons of peripheral cues. Our observations indicate that, in addition to promoting “normal-weight central obesity”, a phenotype associated with adverse metabolic outcomes that is frequently observed in the human population [44,45,60,61], BoNT/B expression in hypothalamic tanycytes induces insulin resistance and glucose intolerance, underscoring the involvement of tanycytes and their transcytotic activity in the pathogenesis of type 2 diabetes.

Our study highlights the crucial role of transcytotic vesicular trafficking in transporting peptide hormones, such as leptin, from the pituitary portal blood circulation to the hypothalamus for maintaining energy homeostasis. However, it is essential to recognize that blocking vesicular transcytotic activity with our current approach may disrupt the transport of various other blood-borne metabolic hormones released by peripheral tissues, which are known to exert an action in the hypothalamus. Therefore, the metabolic phenotype observed in BoNT/B^Tan^ mice likely results from a global impairment of the access of multiple metabolic signals to the brain rather than the specific disruption of a single feedback loop. For instance, while our results demonstrate metabolic alterations in BoNT/B^Tan^ mice, we cannot rule out the possibility that the insulin resistance observed is partially attributable to their increased intra-abdominal adiposity. In addition, BoNT/B may also impair the release of tanycyte-derived signals that play a role in these processes. For example, the impaired regulation of glucose homeostasis in BoNT/B^Tan^ mice might result from the combined alteration of both leptin and insulin transport into the ARH. Notably, when insulin transport into the ARH was impaired by ablating insulin receptors in tanycytes, mice exhibited insulin resistance without changes in glucose tolerance [18]. Conversely, mice with abrogated leptin transport due to the selective knockout of LepR in tanycytes showed increased insulin secretion at early stages despite maintaining normal glucose and insulin tolerance [16]. Remarkably, the absence of both insulin and leptin receptors in ARH POMC neurons, one of the two key neuronal targets of the metabolic hormones transported by tanycytes, resulted in glucose intolerance, insulin resistance, and heightened glucose-stimulated insulin secretion from pancreatic islets [62], a phenotype similar to that observed in BoNT/B^Tan^ mice.

Environmental and age-related changes in tanycyte function can also significantly impact healthy aging. For instance, obese individuals appear to exhibit impaired transport of leptin from the bloodstream into the cerebrospinal fluid [63,64], a condition observed in diet-induced obesity in minipigs [46] and mice [24], as well as in mice with selective knockout of leptin receptors in tanycytes [16]. This points to tanycytes as a prime target of aging processes. Whether this impairment extends to other peptide metabolic hormones remains to be explored. Nevertheless, it is well-established that midlife obesity and diabetes are risk factors for Alzheimer's disease and other forms of dementia later in life [65]. The BoNT/B^Tan^ mouse model, which exhibits alterations in spatial working memory, provides a unique opportunity to investigate the role of tanycytic dysfunction in multiple age-related conditions. This model mimics key features of age-related obesity, such as increased intra-abdominal fat accumulation without significant body weight gain and impaired glucose metabolism. It could therefore prove useful to explore not only whether tanycytic dysfunction contributes to these metabolic changes but also its role in cognitive decline, including dementia, and other hormone-regulated pathological processes associated with aging. Such a mechanism, is especially relevant considering that the bridge formed by tanycytes between the CSF and blood seems to be compromised in Alzheimer's patients [66], and that tanycytes also appear to mediate estrogenic effects on ARH neurons controlling metabolism [67], a finding that could be of particular significance in postmenopausal women who are susceptible to both metabolic dysfunctions and developing Alzheimer's disease.

From a therapeutic point of view, in keeping with the central regulation of peripheral metabolic processes, recent studies have underscored the pivotal role of the brain in the mechanisms of action of emerging anti-obesity and anti-diabetic medications. For instance, our research has recently revealed that hindering tanycytic transcytosis using the same BoNT/B^Tan^ mouse model used in the current study not only impedes the entry of the anti-obesity/anti-diabetic drug liraglutide into the brain but also mitigates its anti-obesity effects on parameters such as food intake, body weight, fat mass, and fatty acid oxidation [17]. Novel drugs combining GLP-1R and glucose-dependent insulinotropic polypeptide (GIP) coagonism are also known to exert their regulatory effects on body weight and food intake through central nervous system pathways [68,69], and it may be possible to enhance their efficacy or specificity by targeting their entry into the brain.

To summarize, our study not only reveals that tanycytic transcytosis is an essential mechanism in the control of metabolic homeostasis by the brain, the blockade of which has far-ranging effects on both central and peripheral processes, providing clues to the pathophysiology of conditions such as type 2 diabetes and obesity as well as age-related cognitive decline, but also validates a tool – the BoNT/B^Tan^ model – that can be used to elucidate these complex and interlinked regulatory processes and design effective therapeutic strategies for the pathologies that result from their breakdown.

## CRediT authorship contribution statement

**Manon Duquenne:** Methodology, Investigation, Conceptualization. **Eleonora Deligia:** Methodology, Investigation, Conceptualization. **Cintia Folgueira:** Investigation. **Cyril Bourouh:** Investigation. **Emilie Caron:** Methodology, Formal analysis. **Frank Pfrieger:** Writing – review & editing, Resources. **Markus Schwaninger:** Writing – review & editing, Funding acquisition. **Ruben Nogueiras:** Writing – review & editing, Funding acquisition. **Jean-Sébastien Annicotte:** Investigation, Funding acquisition. **Monica Imbernon:** Writing – original draft, Investigation, Funding acquisition, Conceptualization. **Vincent Prévot:** Writing – review & editing, Validation, Supervision, Methodology, Investigation, Funding acquisition, Formal analysis, Conceptualization.

## Declaration of competing interest

The authors declare that they have no known competing financial interests or personal relationships that could have appeared to influence the work reported in this paper.

## Figures and Tables

**Figure:**
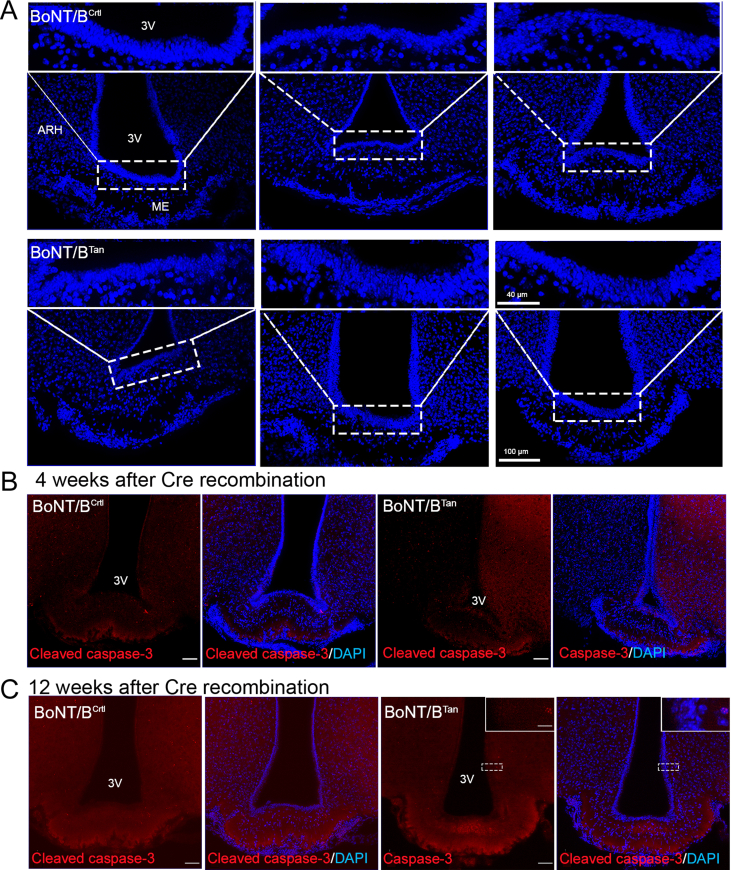
**Supplementary Figure 1**. The expression of Botulinum toxin type B light chain (BoNT/B) in tanycytes does not induce tanycytic cell death. (**A**) Representative images from three BoNT/B^loxP/loxP^ and BoNT/B^Tan^ littermates are presented, captured 12 weeks after mock or Cre-mediated genetic recombination. The top subpanels depict higher magnification of the framed regions in the corresponding bottom panels. (**B–C**) Representative cleaved caspase-3 immunoreactivity in frontal sections of the tuberal region of the hypothalamus from BoNT/B^loxP/loxP^ and six BoNT/B^Tan^ littermates, captured at 4 weeks (**B**, n = 3 per genotype) and 12 weeks (**C**, n = 4 and 6, respectively) after mock or Cre-mediated genetic recombination. Note the absence of cleaved caspase 3 immunoreactivity in cell nuclei lining the floor and wall of the 3V. In **C**, the inset shows a cleaved caspase 3 immunoreactive cell in the parenchyma. Scale bars, 50 μm in **B** and **C** (10 μm in inset).

**Figure:**
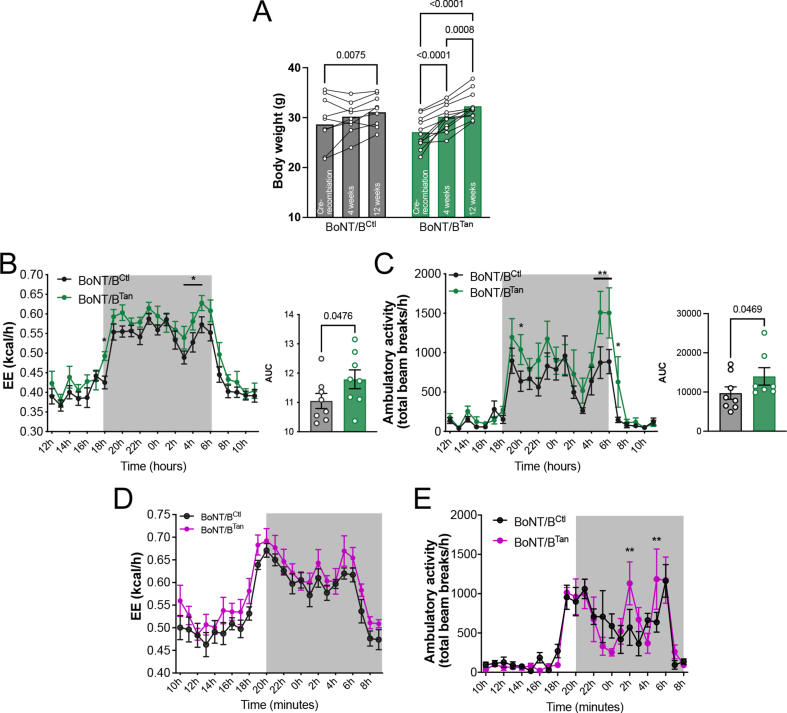
**Supplemenatry figure 2**. Energy expenditure and ambulatory activity in mice fed on chow or high-fat diet. (**A**) Body weight of BoNT/B^Ctl^ (n = 9) and BoNT/B^Tan^ (n = 12) littermates, 4 and 12 weeks after Cre-induced genetic recombination by TAT-Cre infusion into the third ventricle. Values are expressed in grams (gr). Two-way ANOVA, Genotype x time p = 0.0019, F2, 38 = 7.405, Time p < 0.0001, F1, 19 = 0.1438; subjects p < 0.0001, F (19, 38) = 14; Tukey's multiple comparisons test. (**B**–**C**) Curves representing 24h energy expenditure **B** and ambulatory activity **C** in BoNT/B^Ctl^ (n = 7) and BoNT/B^Tan^ (n = 8) mice 4 weeks after the beginning of the experiment (AAV1/2-injected mice) Cre-induced genetic recombination. Two-way ANOVA. In **B**, Interaction p = 0.6239 F _(23, 322)_ = 0.8816; Time p < 0.0001, F _(23, 322)_ = 58.66; Subject p = 0.1029, F _(1, 14)_ = 3,045. LSD post hoc, 18h p = 0.0164; 5h p = 0.0486 and 6h p = 0.0475. AUC, one-tailed Mann Whitney t = 1.789, df = 14. In **C**, Interaction p = 0.0868, F _(23, 299)_ = 1.449; Time p < 0.0001, F _(23, 299)_ = 22.06; Subject p = 0.1383 F _(1, 13)_ = 2.493. LSD post-hoc test, 20h p = 0.0481; 5h p = 0.0018; 6h p = 0.0025 and 7h p = 0.0182. AUC, one-tailed Mann Whitney. (**D**) Curves representing 24 h EE in BoNT/B^Ctl^ (n = 6) and BoNT/B^Tan^ (n = 6) mice at 8 weeks (AAV1/2-injected mice) and 4 weeks of HFD. Two-way ANOVA, Interaction p = 0.9293, F _(23, 230)_ = 1.814; Time p < 0.0001, F _(23, 230)_ = 38.70; Subject p = 0.2078, F _(1, 10)_ = 1.814. LSD post-hoc test. (**E**) Curves representing 24h ambulatory activity in BoNT/B^Ctl^ (n = 6) and BoNT/B^Tan^ (n = 6) mice at 8 weeks (AAV1/2-injected mice) and 4 weeks of HFD. Two-way ANOVA, Interaction p = 0.2022, F _(23, 230)_ = 1.253; Time p < 0.0001, F _(23, 230)_ = 14.16; Subject p = 0.8224, F _(1, 10)_ = 0.05313. LSD post-hoc test, 2.00 p = 0.0064, 5.00 p = 0.0075. Data are expressed as mean ± SEM. p < 0.05 values are indicated in the figures.

## Data Availability

Data will be made available on request.
