# Supplementary material for: Tanycytic transcytosis inhibition disrupts energy balance, glucose homeostasis and cognitive function in male mice
Source: Mol Metab. 2024 Jul 22;87:101996. doi: 10.1016/j.molmet.2024.101996 (PMC11340606; doi:10.1016/j.molmet.2024.101996)
Supplement: Multimedia component 1 [file mmc1.docx]

| Gene | Sense Primer | Antisense Primer |
| --- | --- | --- |
| Slc2a2 | AACCGGGATGATTGGCATGT | GGCGAATTTATCCAGCAGCA |
| Gck | GCTCAGTGAACCCCGGTCAGC | TGTGCGCAGCTGCTCTGAGG |
| Kcnj11 | CACAAGCTGGGTTGGGGGCTC | TGCCCCTCAGCTGGGTTCTGC |
| Glp-1r | GTTTCCTCACGGAAGCGCCA | AAGGAACCTGGGGGCCCATC |
| Ins1 | GCCAAACAGCAAAGTCCAGG | GTTGAAACAATGACCTGCTTGC |
| Pcsk1 | TGATGATCGTGTGACGTGGG | GGCAGAGCTGCAGTCATTCT |
| Pdx1 | ATTGTGCGGTGACCTCGGGC | GATGCTGGAGGGCTGTGGCG |
| MafA | TCCGACTGAAACAGAAGCGG | CTCTGGAGCTGGCACTTCTC |
| Nkx2.2 | GTGCAGGGAGTATTGGAGGC | GAAGGGCCAGAGGAGGAGA |
| Hnf1a | GGTGCGTGTCTACAACTGGT | ACCGTACACCGTGGACCTTA |
| Ucn3 | TGATGCCCACCTACTTCCTG | CTGTGTTGAGGCAGCTGAAG |
| NeuroD1 | CTTGGCCAAGAACTACATCTGG | GGAGTAGGGATGCACCGGGAA |
| ATF4 | ATGGCCGGCTATGGATGAT | CGAAGTCAAACTCTTTCAGATCCATT |
| Xbp1t | GAGCAGCAAGTGGTGGATTT | CCGTGAGTTTTCTCCCGTAA |
| Xbp1s | GAGTCCGCAGCAGGTG | GTGTCAGAGTCCATGGGA |

**Supplementary Table 1***.* Name of the genes of interest and sense and antisense primers sequences used to amplify them as provided by the provider (ThermoFisher).
